# Exploring the Thoughts, Needs and Fears of Chemotherapy Patients—An Analysis Based on Google Search Behavior

**DOI:** 10.3390/healthcare12171689

**Published:** 2024-08-24

**Authors:** Deniz Özistanbullu, Ronja Weber, Maria Schröder, Stefan Kippenberger, Johannes Kleemann, Henner Stege, Roland Kaufmann, Bastian Schilling, Stephan Grabbe, Raphael Wilhelm

**Affiliations:** 1 Department of Dermatology, Venereology and Allergology, University Hospital Frankfurt, Goethe University, 60596 Frankfurt, Germany; 2 Department of Gynecology and Obstetrics, University Mainz, Langenbeckstr. 1, 55131 Mainz, Germany; 3 Department of Dermatology, University Medical Center of the Johannes Gutenberg University, 55131 Mainz, Germany

**Keywords:** chemotherapy, Google Ads, public health, person-centered care, Infodemiology

## Abstract

Chemotherapy poses both physical and psychological challenges for patients, prompting many to seek answers independently through online resources. This study investigates German Google search behavior regarding chemotherapy-related terms using Google AdWords data from September 2018 to September 2022 to gain insights into patient concerns and needs. A total of 1461 search terms associated with “chemotherapy” were identified, representing 1,749,312 to 28,958,400 search queries. These terms were categorized into four groups based on frequency and analyzed. Queries related to “adjuvant” and “neoadjuvant” chemotherapy, as well as “immunotherapy”, suggest potential confusion among patients. Breast cancer emerged as the most searched tumor type, with hair loss, its management, and dermatological issues being the most searched side effects. These findings underscore the role of search engines such as Google in facilitating access to healthcare information and provide valuable insights into patient thoughts and needs. Healthcare providers can leverage this information to deliver patient-centric care and optimize treatment outcomes.

## 1. Introduction

Chemotherapy stands as a cornerstone in the treatment arsenal for numerous cancer types. Despite its routine application in oncological centers worldwide, the utilization of chemotherapy and the burden of the disease itself exact significant physical and psychological tolls on each patient [1]. Chemotherapy-induced symptoms not only diminish patients’ quality of life (QoL) but also evoke existential fear, fatigue and distress across various facets of daily life. Consequently, individualized medical support and guidance are crucial for every patient undergoing chemotherapy. However, even within an environment fostering open dialogue, it cannot be assumed that all patients articulate their needs, fears and thoughts during clinical consultations.

Recently, the internet has emerged as an invaluable resource for health-related information, accessible to a broad demographic and continuously increasing in significance [2,3]. An analysis of passively tracked browsing behavior among German internet users revealed that approximately 85% visited domains associated with health, underscoring the internet’s pivotal role in health information dissemination [4]. Notably, individuals diagnosed with cancer frequently turn to the internet for information, with “cancer” ranking among the most searched health topics on platforms such as Google [5,6].

In Germany, where approximately 95% of citizens over the age of 14 utilize the internet and 80% use it daily, approximately 78% access a search engine at least once a week [7]. Consequently, analyzing internet search behavior among patients and their relatives, either directly or indirectly affected by chemotherapy, offers valuable insights into their collective interests, concerns and fears.

Internet search analysis represents a promising avenue for investigating the population’s engagement with specific topics [8,9,10,11,12]. Widely employed by communication and marketing departments, this methodology has been utilized to examine cancer incidence and mortality rates, identify medical interests, and draw epidemiological conclusions from Google data [13,14,15].

In this investigation, we examined search terms and their corresponding search volumes related to chemotherapy by utilizing the Google AdWords Keyword Planner over a four-year period in Germany. Given Google’s market share dominance in Germany—representing 80% of desktop searches and 96% of all mobile searches—the search volumes derived from this platform are deemed representative [16].

The aim of this study was to elucidate the interest of the German population in chemotherapy by analyzing Google searches related to chemotherapy terms. By doing so, we aimed to glean insights into the thoughts, needs and fears of patients and their relatives, thereby augmenting their medical care.

## 2. Materials and Methods

### 2.1. Google AdWords

Google AdWords, now called Google Ads, is an application developed by Google to support marketing campaigns. It is one of the most used advertising systems on the internet and offers a variety of tools and features to create and optimize advertising campaigns. It enables the determination of monthly search volumes for specific search terms. We used Google AdWords to identify search terms related to the German term for chemotherapy (“Chemotherapie”) over a four-year period. Since Google no longer publishes absolute search numbers but ranges of search frequencies for search terms, we classified the search terms into 4 groups with different search frequencies (1 to 100, 100 to 1000, 1000 to 10,000 and 10,000 to 100,000 searches per month).

### 2.2. Google Trends

Google Trends is a web-based tool provided by Google that allows users to analyze the popularity of specific search terms over a specified period and region. Google Trends aggregates anonymized search data from Google Search to generate data in a normalized rank between 1 and 100 but does not provide information about absolute data. The value 100 represents the highest popularity of the search term. The value 50 means that the term is half as popular, and the value 0 indicates that there were not enough data available for the term. Therefore, it provides insights into the relative search volume for different keywords, helping researchers and marketers to understand the search behavior of internet users.

We used Google Trends to obtain an overview of the search volume development of chemotherapy over the entire four-year study period in Germany.

### 2.3. Statistical Analysis

Standard deviations were calculated using Microsoft Excel Version 16.76. Figures and tables were created with Excel Version 16.76, diagrams.net v24.0.6 and BioRender.com, 2024.

## 3. Results

### 3.1. Chemotherapy Was Consistently Popular throughout the Investigation Period

**Figure 1:**
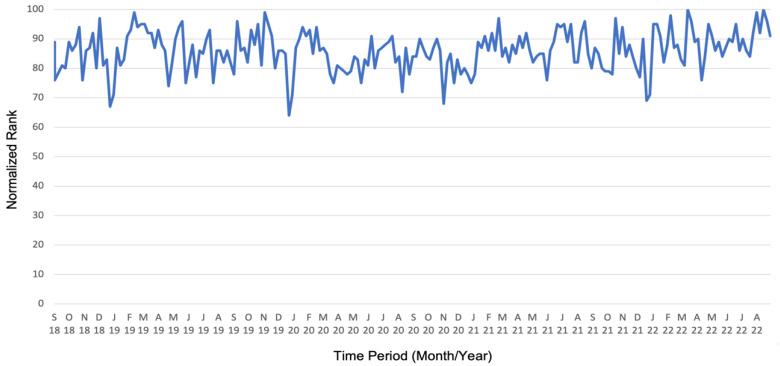
Google Trends analysis of chemotherapy search frequency, showing consistently high ranks and seasonal declines in December and January.

The data analysis of the search frequency of the term chemotherapy using Google Trends is shown in Figure 1. The normalized search rank, which illustrates the search frequency, fluctuates on a high level between 64 and 100. On average, a consistently high normalized rank is observed each year: September 2018 to August 2019: 85.9 ± 7.1; September 2019 to August 2020: 84.7 ± 6.8; September 2020 to August 2021: 85.6 ± 5.9; September 2021 to August 2022: 87.5 ± 7 (mean normalized search rank ± SD). The search frequency declines seasonally in the months of December and January.

### 3.2. General Information, Side Effects and Tumor Types Are Searched Most Frequently

A total of 1461 search terms associated with chemotherapy were found using Google AdWords between September 2018 and September 2022 in Germany. These were categorized into four groups based on their search frequency per month (see Table 1). Overall, our analysis includes 36,444 to 603,3000 search queries per month and a total search volume of 1,749,312 to 28,958,400 search queries.

**Figure 2:**
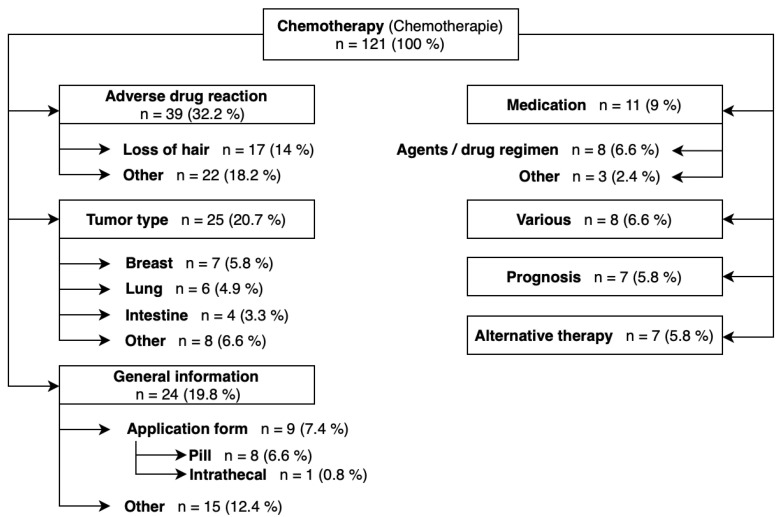
Classification of search terms which are searched for 100 to 1000 times per month.

The 17 most frequently searched terms are shown in Table 2. It is striking that these are primarily general search terms. For example, there are three different search terms for “adjuvant” and “neoadjuvant” chemotherapy with minimal changes to the spelling. In addition to these terms, the common topics of “process”, “side effects” and “success” of chemotherapy were searched very often. Questions are also directed to the search engine: “What is the toughest/strongest chemotherapy?”. Figure 2 illustrates the analysis of the 121 search terms which were searched between 100 and 1000 times per month. Search terms that address general information (19.8% of all 121 search terms), side effects (32.2% of all 121 search terms) and tumor type (20.7% of all 121 search terms) were most frequently searched for. The application form of chemotherapy was the most frequently searched issue of general information (7.4% of all search terms, 37.5% of all general information). By far the most often discussed side effect was hair loss (14% of all 121 search terms, 43.6% of all adverse effects), and the most searched tumor type was breast cancer (5.8% of all search terms, 28% of all tumor types). Of all search terms, 9% related to the medication, and 5.8% each to prognosis and alternative treatment options. “FOLFIRINOX” (folinic acid, 5-fluorouracil, irinotecan, oxaliplatin) and platin derivatives were the drug regimens with the highest search volume. Search terms for alternative therapy were in all cases formulated as a combination of the cancer type and the addition “without chemotherapy” (e.g., “breast cancer without chemotherapy”).

### 3.3. The Specificity of the Search Terms Increases with Decreasing Search Frequency

**Figure 3:**
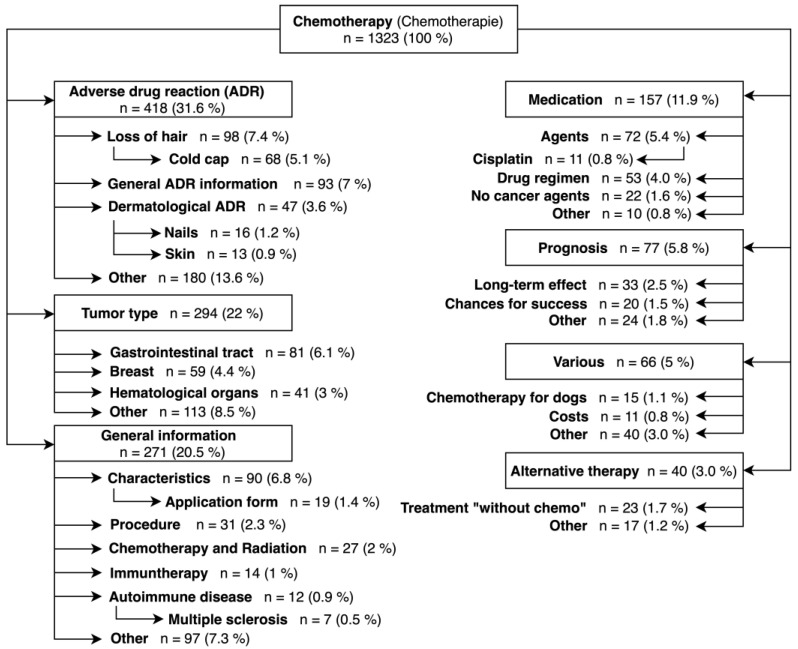
Classification of search terms which are searched for 1 to 100 times per month. Abbreviations: ADR—Adverse Drug Reaction.

Figure 3 shows the analysis of the 1323 search terms searched between 10 and 100 times per month.

Again, drug side effects (31.6% of all search terms), tumor types (22% of all search terms) and general information (20.5% of all search terms) are the most frequently searched categories. The side effect most searched for is hair loss (7.4% of all search terms, 23.4% of all side effects). Of the hair-loss-associated search terms, 68 of 98 (69.3%) address a cold cap. Dermatological side effects represent other side effects often searched for (3.6% of all search terms, 11.2% of side effects).

The most frequent tumor types searched for are gastrointestinal tumors (6.1% of all search terms, 27.6% of all tumor types) and breast cancer (4.4% of all search terms, 20.1% of all tumor types).

Of the search terms, 271 address general information (20.5% of all search terms), and 157 search terms pharmaceuticals (11.9% of all search terms). Here, different chemotherapeutic drugs were searched most frequently (5.4% of all search terms, 45.9% of all pharmaceuticals). Cisplatin (0.8% of all search terms, 7% of all medicines) was searched for most often.

There are 77 search terms that address prognosis (5.8% of all search terms). Here, long-term consequences (2.5% of all search terms, 42.8% of all prognoses) and the success of chemotherapy (1.5% of all search terms, 26% of all prognoses) are most frequently searched for. Search terms that did not fit into the other categories were summarized in “Various” (66 terms in total, 5% of all search terms). Here, chemotherapy for dogs (1.1% of all search terms, 22.7% of all various) and costs of chemotherapy (0.8% of all search terms, 16.7% of all various) were the most popular search queries. Forty search terms address alternative therapies (3% of all search terms). Again, the phrase “without chemo” was the most common (1.7% of all search terms, 57.5% of all alternative therapies).

### 3.4. The Most Frequently Searched Tumor Type Is Breast Cancer

**Figure 4:**
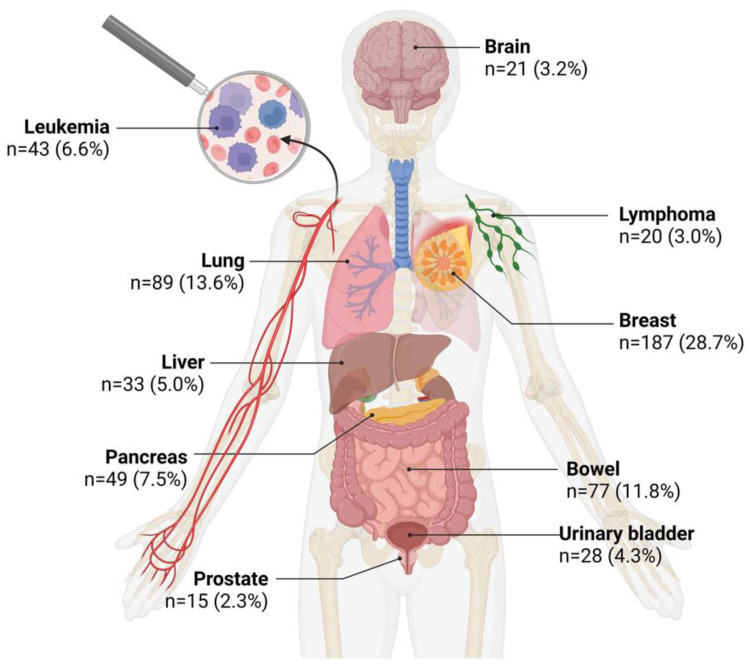
Illustration of the top ten chemotherapy-associated tumor types. Breast cancer is the most frequently searched tumor type. The number of search queries searched 100 to 1000 times per month was scored ten-fold compared to search terms searched 1 to 100 times, which were rated once. Created with BioRender.com.

To evaluate all tumor-type-specific search terms, the search terms from group 3 were scored ten-fold, and those from group 4 once (Figure 4). In groups 1 and 2, were no tumor-type-specific search terms. The most searched cancer is breast cancer (28.7%), which was searched more often than lung cancer (13.6%), prostate cancer (2.3%) and skin cancer (1.4%) together, indicating a notable deviation from the epidemiological prevalence.

### 3.5. Hair Loss Is by Far the Most Searched Side Effect of Chemotherapy

**Figure 5:**
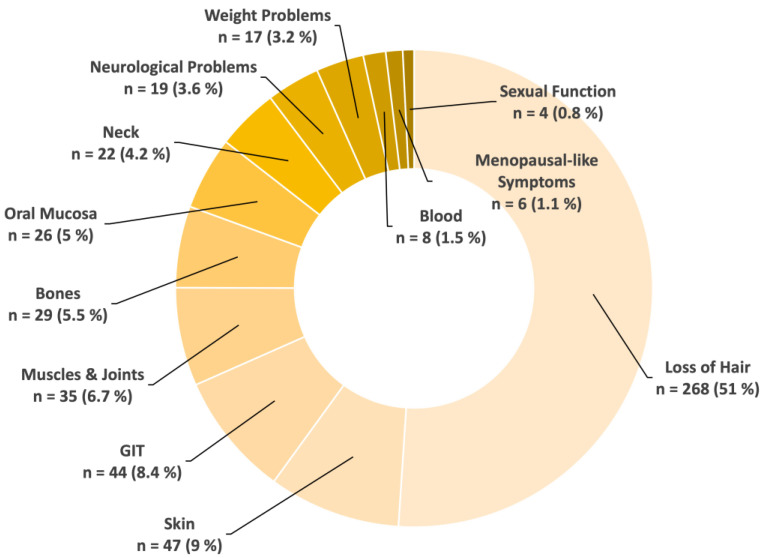
Overview of chemotherapy-associated side effects. Hair loss was the side effect with the highest search volume. The number of search queries that were categorized as searched 100 to 1000 times per month was rated ten-fold compared to search terms searched 1 to 100 times, which were rated once. Abbreviations: GIT—Gastrointestinal Tract.

To quantitatively analyze all search terms associated with side effects, the number of search queries that were categorized as searched 100 to 1000 times per month were again rated ten-fold compared to search terms searched 1 to 100 times, which were rated once (see Figure 5). It turns out that by far the most searched side effect of chemotherapy is hair loss (51%). Skin-related side effects (9%) represented the second most frequently searched side effect.

## 4. Discussion

In this study, we used Google AdWords to offer valuable insights into the preferences and interests of cancer patients and their relatives in Germany by analyzing chemotherapy-related search terms and their corresponding volumes. We aimed to understand the collective interests, concerns and information-seeking behaviors of individuals affected by cancer, either directly as patients or indirectly as relatives.

Infodemiological data are highly relevant in medical research and public health as they provide crucial insights into a population’s interests and identify unknown health issues and trends [15,17]. Google AdWords proves to be a valuable tool for obtaining such data, as it enables the specific analysis of the search behavior over a defined period of time. It is a well-established procedure and was used in many studies [9,10,18,19,20,21,22].

Over the four-year study period, approximately 1.7 to 29.9 million Google searches related to chemotherapy were conducted in Germany.

Notably, Google Trends analysis showed a consistently high search interest with an average search rank of 85.9 throughout the analyzed period [23]. The observed enormous interest mirrors the importance of cancer in Germany, with 497,900 new cancer cases in 2018 [24] and cancer being the second leading cause of death in Germany [25]. It also highlights the enduring significance of chemotherapy in the context of cancer care despite new upcoming treatment modalities. The seasonal fluctuations observed in search frequency, particularly during December and January, may reflect variations in healthcare-seeking behaviors or heightened awareness around cancer-related issues during certain times of the year, such as cancer awareness months or holiday seasons.

Our analysis identified a diverse range of search terms associated with chemotherapy, reflecting the multifaceted nature of patients’ and caregivers’ information needs. General information about chemotherapy processes, side effects and treatment success emerged as predominant themes among the most frequently searched terms.

In particular, chemotherapy was the most searched term since Google AdWords displays not only related search terms but also the search volume of the search term itself. Yet, the frequency of searches for “adjuvant chemotherapy” and “neoadjuvant chemotherapy” is striking and suggests potential confusion between these concepts. Healthcare providers should consider this and precisely define and explain these terms, ensuring that patients understand the therapeutic concept.

The frequent searches for immunotherapy suggest a potential confusion between chemo- and immunotherapy. In context with this search term, we could often see queries such as “Chemo Immunotherapy”. This might suggest that these treatment options are often not thoroughly understood. As immunotherapy is a relatively new therapeutic approach in antineoplastic treatment of specific tumor entities, patients should be educated about the differences between these two options to prevent misunderstandings.

The most frequently searched tumor entity was breast cancer (28.7%), which is also the most common cancer among women, followed by lung cancer (13.6%), colorectal cancer (11.8%), pancreatic cancer (7.5%) and leukemia (6.6%). Interestingly, prostate carcinoma, the most common cancer in men [24], is only rarely searched for (2.3%), even less frequently than lymphomas (3%). One reason for this could be that men generally seek less health-related information [26,27,28,29]. Breast cancer is searched for more than twice as frequently as the second most searched colorectal cancer, signifying a substantial demand for information. Many studies suggest that women use the internet more to acquire health-related information [26,27,28,29,30], which could explain the prevalence of breast cancer searches. All in all, the prominence of certain tumor types, particularly breast cancer, in internet searches highlights the differential information needs and concerns across cancer populations.

Notably, the high prevalence of specific search queries related to chemotherapy side effects underscores the significant impact of treatment-related adverse events on patients’ experiences and perceptions of chemotherapy. Interestingly, 51% of these queries are associated with hair loss or hair-loss-related topics. This is in accordance with previous findings showing chemotherapy-associated hair loss to be one of the most traumatic side effects of chemotherapy [31]. The high search volume for interventions like cold caps suggests a growing interest in supportive care strategies among patients seeking to alleviate treatment-related distress. Therefore, physicians should educate patients about this important side effect and its management prior to treatment start.

Besides the dominance of hair-loss-associated topics, searches for side effects concerning the skin and oral mucosa are also frequent. This highlights an existing high and possibly unmet dermatological information demand.

Interestingly, only few searches address the costs of chemotherapy. This could be attributed to the fact that German health insurance typically covers the costs of chemotherapy. An analysis of the popularity of cost-related queries regarding chemotherapy in other healthcare systems and different demographic and socio-economic groups should be explored in future studies. Furthermore, understanding how socio-economic status affects the usage of digital health in general could help to identify disparities in information accessibility.

Overall, this study underscores the utility of internet search analysis as a complementary approach for understanding the information needs and preferences of cancer patients and their caregivers. By leveraging digital platforms to assess population-level engagement with chemotherapy-related topics, healthcare providers may develop educational resources and supportive care services to enhance patient-centered cancer care and improve health outcomes. Google Ads proves to be a valuable tool in this context. However, several limitations should be acknowledged, including the inability to capture individual motivations or experiences comprehensively. Further, we could not distinguish whether a physician, a relative or a patient searched for a specific term. Hence, we cannot capture only patient interests. In addition, our findings are likely to be limited to the search behavior of Google users in Germany, cannot be directly applied to other countries and may not fully capture the experiences of individuals with limited access to the internet or those with intellectual disabilities. For future research, it is crucial to explore how such barriers affect information-seeking behaviors and to develop strategies to include these populations in digital health studies. Replicating this study with additional methodologies, such as qualitative interviews or surveys targeting populations with restricted internet access, could provide a more comprehensive understanding of the informational needs across diverse groups. Additionally, we cannot compare exact numbers of search queries, as only ranges of searches are provided by Google AdWords, which restricts the precision of our analysis and makes it impossible to perform detailed statistical comparisons based on exact counts. Moreover, there is a lack of information on age, gender and other characteristics of the individuals who used Google. The impact of Google advertising on search behavior also remains unexplored. Future research should explore integrated approaches combining quantitative internet search analysis with qualitative methods to gain a more nuanced understanding of patients’ information-seeking behaviors and support needs throughout the cancer journey.

## 5. Conclusions

Overall, our findings underscore the ongoing need for accessible information and support resources to address the physical and psychological burdens associated with chemotherapy.

Internet search analysis proves to be a valuable approach for understanding the information needs and preferences of cancer patients. Our findings reveal potentially unmet needs related to medical information and highlight the importance of providing comprehensive educational resources that address not only the technical aspects of treatment but also the potential impacts on patients’ quality of life and overall well-being.

## Figures and Tables

**Table 1:** Overview of the four different groups, their number of search terms and word count. Word counts are shown ± SD.

	Total Searches	*n*	Word Count per Search Term
Group 1	10,000 to 1,000,000	2	1.5 ± 0.7
Group 2	1000 to 10,000	15	2.3 ± 1.1
Group 3	100 to 1000	121	2.6 ± 1.0
Group 4	1 to 100	1.323	2.8 ± 0.8

**Table 2:** All searched keywords of Group 1 and Group 2 in German language and their corresponding English translation.

Keyword	Corresponding English Term	Group
Chemo Therapie	Chemotherapy	1
Chemotherapie	Chemotherapy	1
Adjuvant Chemotherapie	Adjuvant Chemotherapy	2
Adjuvante Chemotherapie	Adjuvant Chemotherapy	2
Adjuvanten Chemotherapie	Adjuvant Chemotherapy	2
Chemo Nebenwirkungen	Side effects of Chemotherapy	2
Chemotherapie Ablauf	Chemotherapy regime	2
Chemotherapie Erfolgsquote	Success rate of Chemotherapies	2
Chemotherapie Nebenwirkungen	Side effects of Chemotherapy	2
FOLFIRINOX	FOLFIRINOX	2
Nebenwirkungen Chemotherapie	Side effects of Chemotherapy	2
Neoadjuvant Chemotherapie	Neoadjuvant Chemotherapy	2
Neoadjuvante Chemotherapie	Neoadjuvant Chemotherapy	2
Neoadjuvanten Chemotherapie	Neoadjuvant Chemotherapy	2
Palliative Chemotherapie	Palliative Chemotherapy	2
Welche Chemo ist am schlimmsten	What is the toughest Chemotherapy	2
Welche Chemo ist die stärkste	What is the strongest Chemotherapy	2

## Data Availability

The original contributions presented in the study are included in the article; further inquiries can be directed to the corresponding author.
